# Downregulation of serum vitamin D receptor level, serum 25-hydroxyvitamin D, and association of vitamin D receptor gene polymorphisms ApaI and TaqI with obesity in the Bangladeshi population

**DOI:** 10.1371/journal.pone.0337523

**Published:** 2025-11-20

**Authors:** Annur Ferdous, Munira Jahan Raisa, Md Hijbullah, Nafiz Imtiaz Siam, Shatabdy Barua Trisha, Sadia Biswas Mumu, Md Aminul Haque, Javed Ibne Hasan, Muhammed Mahfuzur Rahman, Md Shaki Mostaid

**Affiliations:** 1 Department of Pharmaceutical Sciences, Faculty of Health and Life Sciences, North South University, Dhaka, Bangladesh; 2 Department of Pharmacy, Brac University, Dhaka, Bangladesh; FIOCRUZ, BRAZIL

## Abstract

**Background/Objectives:**

Obesity is a chronic metabolic disorder, and its prevalence in Bangladesh is increasing at an alarming rate. Previous reports have suggested a significant association between Vitamin D receptor (VDR) gene polymorphisms and obesity, but with inconsistent results. The purpose of our study was to investigate the association between two single-nucleotide polymorphisms (SNPs) (Apal, rs7975232, and Taql, rs731236) of the VDR gene and the risk of obesity in the Bangladeshi population. Moreover, we looked at serum VDR levels and serum 25-hydroxy vitamin D levels in people with obesity (n = 124) and healthy controls (n = 126).

**Methods:**

Genotyping was performed using Polymerase Chain Reaction-Restriction Fragment Length Polymorphism (PCR-RFLP). General linear model and multivariate logistic regression analysis were used to calculate the adjusted odds ratio (OR) along with 95% confidence intervals (CI) and P-values.

**Results:**

Serum VDR level was downregulated in people with obesity compared to healthy controls (P < 0.0001) along with significantly lower levels of 25-hydroxy vitamin D (P < 0.0001). For the ApaI rs7975232 (C > A) polymorphism, the CA Heterozygous genotype carried a 1.93-fold higher risk of developing obesity (OR=1.93, 95% CI = 1.10–3.41, P = 0.023). On the contrary, for TaqI, rs731236 (T > C), no significant association was found for both heterozygous and mutant homozygous genotypes.

**Conclusion:**

We report the downregulation of serum VDR levels and serum 25-hydroxy vitamin D levels in people with obesity. Moreover, a polymorphism of Apal (rs7975232 C > A) in the VDR gene increases the risk of developing obesity in the Bangladeshi population.

## Introduction

Obesity is a serious metabolic disorder characterized by a body mass index (BMI) of ≥ 30 kg/m^2^ [1]. It is characterized by the formation of excessive body fat as a result of a metabolic imbalance between energy intake and expenditure [2]. Obesity is a major risk factor for hypercholesterolemia, cardiovascular disorders, diabetes, and some types of malignancy [3]. According to a recent report from the World Obesity Federation (WOF), around one billion people worldwide, one in five women and one in seven men, will be affected by obesity by 2030 [10]. A survey conducted among the Bangladeshi population in 2018 reported that the prevalence of obesity is 5.4% among adults, and obesity prevalence is higher in females (8.6%) compared to males (2.3%) [4,5]. The increased number of people with obesity adds to the growing burden of non-communicable diseases in a developing country like Bangladesh [6,7]. Rapid urbanization and the increase in consumption of high-calorie fast food diets have also contributed to this problem, at least in the young population living in urban areas [8].

The complex etiology of obesity involves both genetic and environmental factors [9] and single-nucleotide polymorphisms (SNPs) in various genes have been associated with obesity [10,11]. It has been suggested that 40% of the etiology of obesity is due to genetic factors. Low level of vitamin D is a risk factor for obesity and play a vital role in its pathogenesis [12]. Several studies have reported a significant relationship between vitamin D deficiency and obesity [13,14]. Vitamin D is one of the fat-soluble vitamins that is obtained from sunlight exposure and dietary intake [2,15]. The ultraviolet light converts 7-hydrocholesterol present in the skin into inactive vitamin D precursors, and two hydroxylation processes in the liver and kidney transform this precursor into the active form calcitriol [2,16,17]. Vitamin D receptor (VDR) is a transcription factor present in adipocytes, and it mediates the action of vitamin D [18]. Multiple studies have reported an association between several genetic variants of the VDR gene with obesity but the results have been contradictory [19,20]. In particular, the polymorphism named ApaI (rs7975232) and TaqI (rs731236) of the VDR gene have been extensively studied and shown a positive association with obesity in Saudi, Iranian, Korean, Greek, and Chinese populations [16,21–26] whereas negative associations have also been reported [16,23,27–29]. No study has investigated the association of ApaI and TaqI polymorphisms of the VDR gene with obesity in the Bangladeshi population.

**Fig 1:**
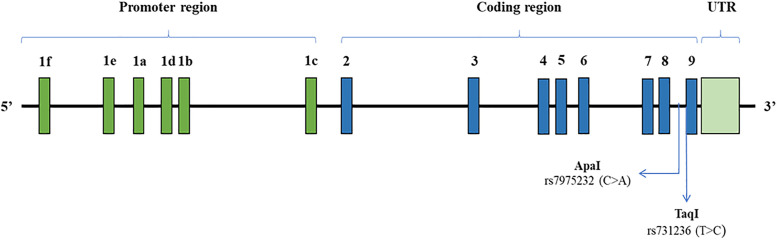
Structure of the VDR gene and position of ApaI (intron 8) and TaqI position (in the coding sequence, exon 9) near the 3’ terminal. UTR = Untranslated region.

Vitamin D levels below 20 ng/ml are considered deficient, 21–29 ng/ml are regarded as insufficient, and 30–100 ng/ml are considered adequate [30]. The body’s photosynthesis and bioavailability of vitamin D are influenced by several factors, including the amount and timing of sun exposure, latitude, season, atmospheric pollution, clothing style, use of sunblock, skin pigmentation, obesity, and the presence of several chronic diseases [31]. Most of these factors also increase the risk of developing an impaired vitamin D level [32,33]. In addition, several studies have shown that 25-hydroxy vitamin D deficiency is strongly associated with obesity in children and adolescents from different ethnicities [15,34]. Overexpression of VDR leads to reduced energy expenditure and results in obesity in mice models [35,36]. VDR gene is located on chromosome 12 and consists of a series of putative polymorphisms, among which ApaI is located in Intron 8 and TaqI on Exon 9 (Fig 1) [37–39].

The purpose of this study was to investigate serum VDR levels and serum 25-hydroxy vitamin D level in those with obesity and healthy controls. Moreover, we examined the association of the VDR gene polymorphisms ApaI (rs7975232) and TaqI (rs731236) with obesity. Additionally, we explored whether rs7975232 and rs731236 exert any cis-regulatory effects on serum vitamin D receptor levels.

## Materials and methods

### Study subjects

This study comprised 250 unrelated individuals, 124 persons with obesity, and 126 healthy controls without obesity from Bangladesh. The sample size was calculated a priori using the G* power software [38]. For an effect size of d = 0.5 (medium effect), α = 0.05, and the samples needed in each group were 125. In this study, BMI, the most widely used parameter, was used as a standard parameter to classify obesity. However, other parameters like waist circumference, waist to hip ratio, and body fat percentage are also widely used specifically for abdominal obesity. The cohorts were classified into people with obesity and healthy control groups according to their body mass index (BMI), where those with BMI ≥ 30 were considered as people with obesity, and BMI < 25 were considered healthy control subjects without obesity [16].

Face-to-face interviews were conducted to get information on smoking, drinking, fast food intake (meals/week), and physical activity with other relevant demographic variables and the participants were asked to fill out a questionnaire. Height, and weight were measured by two independent researchers. The average height and weight were used for the calculation of BMI (BMI = Weight/Height^2^ (kg/m^2^) [39].

The subject inclusion criteria were the absence of acute and chronic diseases, and aged between 18–65 years. The exclusion criteria were the presence of diabetes, hepatic or renal disease, cardiovascular disease, hyperthyroidism or hypothyroidism, and any other disorder or medication affecting body weight. The diagnosis for the diseases considered in exclusion criteria were accessed through the questionnaire answers. The participants were informed about the study objectives, and written informed consent forms were collected. Ethical permission was obtained from the Institutional Review Board (IRB) of North South University (2022/OR-NSU/IRB/1206). The study was carried out in accordance with the Declaration of Helsinki and its subsequent revisions [40].

### Blood sample collection

After overnight fasting, 3 ml of venous blood samples were collected in K_3_-EDTA tubes (BD Vacutainer® blood collection tubes, Becton and Dickinson and Company, USA) and 2 ml was collected in serum separation tubes by trained professional nurses using an aseptic technique. Samples were stored at −80°C until further analysis.

### Genomic DNA extraction and genotyping

Genomic DNA was extracted using the FavorPrep™ Blood Genomic DNA Extraction Kit (Favorgen Biotech Corp. Taiwan) following the manufacturer’s protocol. The DNA concentration was checked using a nanodrop spectrophotometer (Thermo Fisher Scientific, Waltham, MA, USA) and stored in TE buffer until further analysis. Genomic DNA was amplified, and the presence of ApaI (C > A, rs7975232) and TaqI (T > C, rs731236) variants was identified by Polymerase Chain Reaction-Restriction Fragment Length Polymorphism (PCR-RFLP). The primer sequence, SNP position, PCR product length, and expected restriction fragment lengths are mentioned in Table 1.

**Table 1:** Sequence of forward (F) and reverse (R) primers for ApaI (rs7975232) and TaqI (rs731236) in VDR genotyping.

SNP (dbSNP)	Position	Alleles	Primer (5’-3’)	PCR product	Annealing temp (°C)	Restriction fragment length (bp)
**ApaI** *(rs7975232)*	Intron 8	C > A	**F:** AGCAGAGCAGAGTTCCAAGC **R:** GTGAGGAGGGCTGCTGAGTA	745	60	C: 745 A: 528, 217
**TaqI** *(rs731236)*	Exon 9	T > C	**F:** AGCAGAGCAGAGTTCCAAGC **R:** GTGAGGAGGGCTGCTGAGTA	740	60	T: 495, 245 C: 290, 245, 205

**Fig 2:**
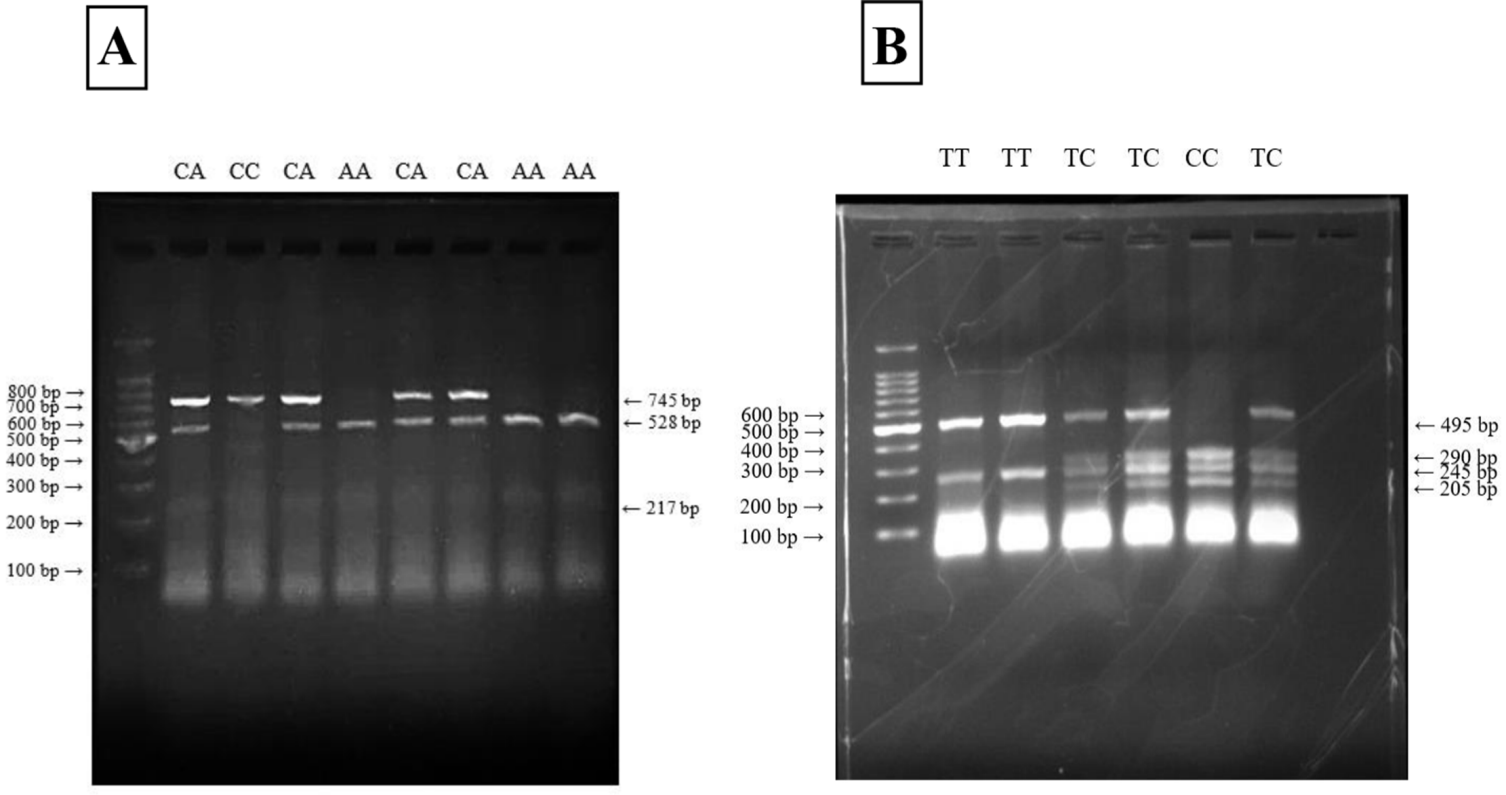
Digestion of the PCR product with restriction enzymes (A) After digesting the PCR product with ApaI restriction enzyme, 745 bp fragments (homozygous genotype CC), partially digested 745, 528, and 217 bp fragments (heterozygous genotype CA), or completely digested 528 and 217 bp fragments (mutant homozygous genotype AA) are present (The 217 bp fragment is not visible in the figure 2A). **(B)** After digesting the PCR product with TaqI restriction enzyme, digested 495 and 245 bp fragments (homozygous genotype TT), partially digested 495, 290, 245, and 205 bp fragments (heterozygous genotype TC), or completely digested 290, 245, and 205 bp fragments (mutant homozygous genotype CC) are present.

The PCR was conducted in a 20 µL reaction using a PCR master mix (Promega, USA) for both ApaI (C > A, rs7975232) and TaqI (T > C, rs731236). The PCR protocol began with an initial denaturation step at 94°C for 4 minutes. This was followed by 36 amplification cycles, each consisting of three distinct phases: denaturation at 94°C for 30 seconds, annealing at 60°C for 30 seconds, and extension at 72°C for 1 minute. Next, a final extension step was performed at 72°C for 5 minutes. After PCR, the lengths of the amplicons were checked by agarose gel electrophoresis (Axygen® Gel Documentation System, Corning, USA). The amplicons were then digested by their respective enzymes. The restriction digestion reaction to determine the genotype of the ApaI variant was conducted in a reaction volume of 20 µL. The mixture comprised 10 µL of PCR product, 2 µL of 10X NEB buffer, 1 µL of ApaI restriction enzyme (New England Biolabs, USA), and nuclease-free water added to a final volume of 20 µL. The reaction was incubated at 37°C for 15 minutes. Post-digestion, the products were resolved on a 1.5% agarose gel, producing genotype-specific patterns: a single 745 bp fragment for the CC (homozygous) genotype, three fragments of 745 bp, 528 bp, and 217 bp for the CA (heterozygous) genotype, and two fragments 528 bp and 217 bp—for the AA (mutant) genotype (Fig 2). 100 bp molecular ladder (Promega, USA) was used to confirm the size of the fragments on the gel.

For TaqI digestion, the reaction was also performed in a 20 µL mixture, including 10 µL of PCR product, 1 µL of TaqI restriction enzyme (TaKaRa Shuzo Co., Japan), 2 µL of BSA (Bovine Serum Albumin), 2 µL of 10X TaqI buffer, and nuclease-free water to adjust the volume. The mixture was incubated at 65°C for 1 hour. Gel electrophoresis revealed genotype-specific patterns: TT (homozygous) displayed two fragments of 495 bp and 245 bp, CC (mutant) showed three fragments of 290 bp, 245 bp, and 205 bp, while TC (heterozygous) exhibited four fragments—495 bp, 290 bp, 245 bp, and 205 bp (Fig 2B). For quality control purposes, 10% of the samples were randomly selected and genotyped, and no discrepancies were found.

### Measurement of serum 25-hydroxyvitamin D level

The levels of 25-hydroxyvitamin D from fasting blood samples were measured by Elecsys^®^ (Cobas, Roche Diagnostics Limited, Switzerland) vitamin D total assay kits using the manufacturer’s protocol. This procedure employs an electrochemiluminescence assay to measure the 25-hydroxyvitamin D level in vitro. Samples were run in duplicate, and the average was taken as the vitamin D level.

### Quantification of serum vitamin D receptor (VDR) level

The VDR level in serum was quantified using a Human Vitamin D Receptor (VDR) ELISA kit (catalog no: SG-10743, SinoGeneclon Biotech Co. Ltd, China) using the manufacturer’s guidelines. Briefly, samples were diluted 5 times using sample diluent and 50 µl of standards, and samples were added in duplicate to their designated wells and allowed to incubate for 30 minutes at 37°C. Then, the liquids were discarded, and the well was washed 5 times with wash buffer. Next, HRP-conjugate reagent was added to the wells (except blank) to form an antigen-antibody-enzyme complex. After thorough washing, TMB substrate solutions were added to the wells and incubated for 15 minutes at 37°C in the dark to form a blue color. Next, a stop solution was added to terminate the reaction, and absorbance was measured at 450 nm. The standard curve was constructed using a four-parameter logistic curve fit, and sample concentration was determined using the standard curve. Finally, the concentration of the samples was determined by multiplying by the dilution factor.

### Statistical analysis

Two-tailed tests were used for all statistical analyses. For categorical variables, the chi-square test was used, while the independent samples t-test was used to compare continuous variables. The chi-square test was also used to find the deviation from the Hardy-Weinberg equilibrium for the genotypes in each group. Multivariate logistic regression was used to see the difference in genotypes between people with obesity and healthy control groups adjusting for age, and sex. Adjusted odds ratio with 95% confidence intervals and P-values were calculated to interpret the findings. P < 0.05 was considered significant.

The Shapiro-Wilk test was used to test the normality of the distribution of serum vitamin D receptor level and serum 25-hydroxy vitamin D levels in cases and controls. A general linear model (GLM) was used to compare the serum vitamin D receptor levels and serum 25-hydroxy vitamin D levels between cases and controls. Data analysis was performed in SPSS version 25. Graphs were prepared by GraphPad Prism 9.

### Expression quantitative test loci analysis

General linear models (GLMs) were used to find the cis-regulatory effects of the SNPs on serum 25-hydroxy vitamin D and vitamin D receptor (VDR) levels. Each model included genotype, case status, and genotype x case status as covariates. Significant genotype x case status interactions were analyzed post-hoc by case status stratification analysis. P < 0.05 was regarded as significant.

## Results

### Characteristics of the study population

Table 2 presents the demographic and clinicopathological characteristics of the study participants.

**Table 2:** Distribution of demographic and clinicopathological characteristics of people with obesity and control group.

Characteristics		People with obesity n = 124(%)	Control Group n = 126(%)	ꭓ^2^ -value	t-value	P-value
Age		40.16 ± 12.84	36.83 ± 15.11	–	−1.88	0.061
Body mass index (BMI)		35.14 ± 4.27	20.46 ± 3.76	–	28.55	**<0.001**
Sex	Female Male	65 (57.0) 59 (43.4)	49 (43) 77 (56.6)	4.612	**–**	**.032**
Fast-food intake (1–3 meals/week)	Yes No	122 (98.4) 2 (1.6)	120 (95.2) 6 (4.8)	2.001	–	.157
Smoking	Smoker Nonsmoker	34 (52.3) 90 (48.6)	31 (47.7) 95 (51.4)	0.25	–	.612
Physical Exercise (30–60 minutes/day)	Yes No	34 (35.1) 90 (58.8)	63 (64.9) 63 (41.2)	13.42	**–**	**<0.001**
Presence of Hypercholesterolemia	Yes No	42 (91.3) 82 (40.2)	4 (8.7) 122 (59.8)	39.22	**–**	**<0.001**
Presence of Hypertension	Yes No	45 (65.2) 79 (43.6)	24 (34.8) 102 (56.4)	9.29		**.002**
Family history of obesity	Yes No	85 (55.6) 39 (40.2)	68 (44.4) 58 (59.8)	5.59		**.018**
Serum Vitamin D	Deficient	81 (65.3)	6 (4.8)	142.7	**–**	**<0.001**
	Insufficient	35 (28.2)	16 (12.7)	45.6	**–**	**<0.001**
	Sufficient	8 (6.45)	104 (82.6)	–	**–**	**–**
Total cholesterol (mmol/L)		6.16 ± 0.98	5.15 ± 0.86	–	−8.68	**<0.001**
Triglyceride (mmol/L)		1.96 ± 0.19	1.17 ± 0.46	–	−4.07	**<0.001**
HDL-C (mmol/L)		1.37 ± 0.44	1.45 ± 0.41	–	1.67	0.097
LDL-C (mmol/L)		3.31 ± 0.71	2.86 ± 0.95	–	−4.22	**<0.001**

Significant values (p < 0.05) are marked in bold.

Hypertension was significantly more common in the people with obesity group (65.2%) compared to the healthy control group (34.8%) (P = 0.002). Additionally, a lack of physical exercise (30–60 minutes/day) was more frequent among individuals with obesity (58.8%) than in the control group (41.2%), showing a strong statistical significance (P < 0.001). Lastly, the family history of obesity also demonstrated a significant difference (P = 0.018), with 55.6% of individuals with obesity reporting a positive family history, compared to 44.4% in the control group.

### Analysis of genotype frequencies of rs7975232 and rs7321236 of the VDR gene

Table 3 presents the allelic and genotypic frequency distribution for the investigated SNPs in both cases and healthy controls. For ApaI, the CA heterozygous genotype conferred a 1.93-fold higher risk of developing obesity compared to healthy controls (adjusted OR=1.93, 95% CI = 1.09–3.41, P = 0.023). In contrast, the AA mutant genotype showed no statistically significant association. For TaqI, there was no statistically significant association for both heterozygous as well as mutant homozygous genotypes.

**Table 3:** Genotype frequencies of ApaI (C > A, rs7975232) and TaqI (T > C, rs731236) polymorphisms in people with obesity and controls.

	Genotypes	People with obesity n = 124(%)	HWEχ^2^ (P)	Control Group n = 126(%)	HWE χ^2^ (P)	Adjusted odds ratio	95% Cl	P
rs7975232	CC CA AA CA + AA A allele	42 (33.9) 57 (46.0) 25 (20.2) 82 (66.1) 107 (38.3)	0.50 (0.48)	32 (25.4) 84 (66.7) 10 (7.9) 94 (74.6) 104 (36.9)	17.75 (<0.05)	ref 1.93 0.52 1.50 1.07	ref 1.09-3.41 0.22-1.24 0.87-2.59 0.75-1.54	ref **0.023** 0.145 0.143 0.424
rs7321236	TT TC CC TC + CC C allele	50 (49) 61 (48.4) 13 (59.1) 74 (50) 87 (35.08)	0.37 (0.44)	52 (51) 65 (51.6) 9 (40.9) 74 (50) 83 (32.92)	0.06 (0.07)	ref 1.03 0.67 0.96 1.10	ref 0.61-1.73 0.26-1.69 0.58-1.59 0.76-1.59	ref 0.927 0.393 0.879 0.613

Significant values (p < 0.05) are marked in bold.

HWE: Herdy-Weinberg equilibrium.

### Association of VDR polymorphisms and clinicopathological characteristics in patients

The analysis of people with obesity and healthy control groups with varied clinicopathological features in Tables 4 and 5 revealed no significant relationship for both ApaI (C > A, rs7975232) and TaqI (T > C, rs731236) polymorphisms, respectively (Tables 4 and 5).

**Table 4:** Correlation of rs7975232 polymorphism with clinicopathological characteristics in people with obesity and healthy controls.

Characteristics		rs7975232 Carrier n = 167(%)	rs7975232 Non- carrier n = 83(%)	Odds ratio (OR)	95% CI	P-value
Sex	Female Male	77 (67.5) 90 (66.2)	37 (32.5) 46 (33.8)	0.940 1.064	0.554-1.596 0.627-1.805	0.819 0.819
Fast-food intake (1–3 meals/week)	No Yes	5 (3.0) 162 (97.0)	3 (3.6) 80 (96.4)	ref 0.87	ref 0.20-3.85	– 0.864
Smoking	Non-smoker Smoker	124 (74.3) 43 (25.7)	61 (73.5) 22 (26.5)	ref 1.05	ref 0.57-1.95	– 0.857
Physical Exercise (30–60 minutes/day)	No Yes	109 (65.3) 58 (34.7)	44 (53.0) 39 (47.0)	ref 1.53	ref 0.85-2.74	– 0.154
Presence of hypercholesterolemia	No Yes	133 (79.6) 34 (20.4)	71 (85.5) 12 (14.5)	ref 0.80	ref 0.34-1.87	– 0.609
Presence of hypertension	No Yes	119 (71.3) 48 (28.7)	62 (74.7) 21 (25.3)	ref 0.96	ref 0.48-1.92	– 0.919
Family history of obesity	No Yes	61 (36.5) 106 (63.5)	36 (43.4) 47 (56.6)	ref 0.92	ref 0.50-1.67	– 0.790

Carrier- Presence of at least one mutant allele or both.

Non-carrier- Absence of mutant allele.

Significant values (p < 0.05) are marked in bold.

**Table 5:** Correlation of rs731236 polymorphism with clinicopathological characteristics of the people with obesity and healthy controls.

Characteristics		rs731236 Carrier n = 148(%)	rs731236 Non- carrier n = 102(%)	Odds ratio (OR)	95% CI	P-value
Sex	Female Male	65 (57) 83 (61)	49 (43) 53 (39)	1.181 0.847	0.712-1.959 0.511-1.405	0.520 0.520
Fast-food Intake (1–3 meals/week)	No Yes	3 (37.5) 145 (59.9)	5 (62.5) 97 (40.1)	Ref 0.401	Ref 0.094–1.718	– 0.219
Smoking	Non-smoker Smoker	110 (59.5) 38 (58.5)	75 (40.5) 27 (41.5)	Ref 1.09	Ref 0.59–1.99	– 0.776
Physical Exercise (30–60 minutes/day)	No Yes	91 (59.5) 57 (58.8)	62 (40.5) 40 (41.2)	Ref 1.02	Ref 0.56–1.83	– 0.961
Presence of Hypercholesterolemia	No Yes	124 (60.8) 24 (52.2)	80 (39.2) 22 (47.8)	Ref 0.906	Ref 0.41–2.02	– 0.809
Presence of Hypertension	No Yes	115 (63.5) 33 (47.8)	66 (36.5) 36 (52.2)	Ref 1.865	Ref 0.96–3.64	– 0.067
Family history of Obesity	No Yes	61 (62.9) 87 (56.9)	36 (37.1) 66 (43.1)	Ref 1.268	Ref 0.70–2.29	– 0.433

Carrier- Presence of at least one mutant allele or both.

Non-carrier- Absence of mutant allele.

Significant values (p < 0.05) are marked in bold.

### Serum 25-hydroxyvitamin D level

**Fig 3:**
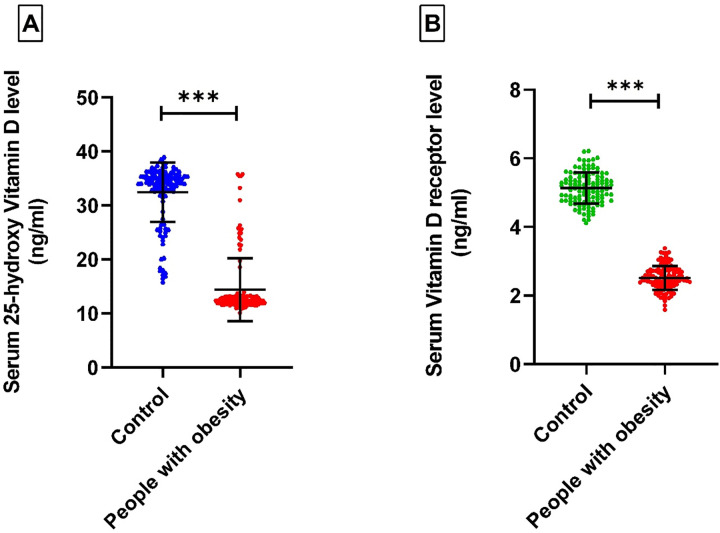
Levels of serum 25-hydroxy vitamin D and serum VDR levels in people with obesity and healthy controls. ***P < 0.0001.

Individuals with obesity were found to have much lower serum 25-hydroxyvitamin D levels compared to healthy individuals (Fig 3A). The average serum 25-hydroxy vitamin D level in the people with obesity group was 14.40 ± 5.80 ng/ml, while the healthy group had a higher average of 32.44 ± 5.49 ng/ml. General linear model analysis showed this difference to be highly significant (P < 0.0001).

### Serum vitamin D receptor (VDR) levels

The mean serum vitamin D receptor (VDR) levels in the people with obesity group were 2.51 ± 0.35 ng/ml, while the control group exhibited higher levels, with a mean of 5.14 ± 0.45 ng/ml (Fig 3B). Statistical analysis using the general linear model revealed a highly significant difference (P < 0.0001).

### Expression quantitative trait loci analysis

Examination of the ApaI (C > A, rs7975232) and TaqI (T > C, rs731236) SNPs with serum 25-Hydroxy vitamin D and VDR level did not reveal any significant results (Table 6).

**Table 6:** Expression of quantitative trait loci analysis.

VDR SNPs	Serum 25-hydroxy vitamin D level F value (P value)	Vitamin D receptor (VDR) level F value (P value)
ApaI (C > A, rs7975232)	0.158 (0.696)	0.012 (0.914)
TaqI (T > C, rs731236)	0.501 (0.489)	2.783 (0.115)

Significant values (p < 0.05) are marked in bold.

Using a general linear model, in terms of serum 25-Hydroxy vitamin D the F value for ApaI (C > A, rs7975232) was 0.158 (P = 0.696), and for TaqI (T > C, rs731236), it was 0.501 (P = 0.48) and for VDR level the F value for ApaI (C > A, rs7975232) was 0.012 (P = 0.914), and for TaqI (T > C, rs731236), it was 2.783 (P = 0.115).

## Discussion

The aim of our study was to evaluate the association between VDR gene polymorphism ApaI (rs7975232, C > A) and TaqI (rs731236, T > C) with the risk of obesity in the Bangladeshi population. Our investigation revealed that polymorphism at rs7975232 was significantly associated with an increased risk of obesity. Serum VDR and 25-hydroxy vitamin D levels were downregulated in people with obesity. Additionally, we observed that females were more prone to become affected by obesity, along with people with a family history of obesity. Moreover, the prevalence of hypertension and hypercholesterolemia was higher in those with obesity compared to controls.

The nuclear vitamin D receptor (VDR), which binds to the active form of 1,25-dihydroxyvitamin D3, plays a crucial role in mediating various biological processes, including the regulation of cellular proliferation, differentiation, and calcium homeostasis [41,42]. Polymorphisms in the VDR gene, particularly rs7975232, have been linked to an increased risk of cardiovascular diseases, hypertension, type 2 diabetes mellitus, and colorectal cancer [24,41–44]. In our analysis, we found that polymorphism at rs7975232 (C > A) conferred 1.93 times more risk of developing obesity in the Bangladeshi population. Our findings are in agreement with a previous case-control study, which reported that people with a CA heterozygous genotype had a higher risk for obesity in the Iranian population [23]. Other studies revealed that the mutant homozygous genotype, AA of ApaI showed an association with the risk of obesity in Chinese children aged 6–14 years and the homozygous genotype, CC of ApaI is responsible for lower serum 25-hydroxy vitamin-D levels in both sexes of Lebanese young population aged 18–30 years [45,46]. Additionally, a study in the Iranian population showed that the A allele of the rs7975232 was associated with an increased risk of obesity [23]. Abouzid et. al described obesity as more common in individuals with at least one A allele [43]. On the contrary, studies in people with obesity from the Saudi male population and the Caucasian population revealed no significant association of the heterozygous genotype of this SNP with the risk of developing obesity [16,27]. In addition, a study that included 131 Saudi female students found a protective effect of the minor A allele of rs7975232 against obesity [22]. The role of the rs7975232 (ApaI) heterozygous genotype may be different in populations of different ethnicities. Further studies need to be conducted to elucidate the effect of this SNP on obesity in different ethnic groups.

For rs731236 (TaqI), we observed no significant association with obesity in the Bangladeshi population. Our results are consistent with several genetic epidemiological studies that reported polymorphism of rs731236 (TaqI) imparts no significant association with the risk of developing obesity in populations from Iran, Bahrain, and Spain [23,28,29]. On the contrary, findings from other case-control studies with Saudi men, the Greek, and the Chinese population showed a significant association of rs731236 with obesity [16,25,26]. Future studies with larger samples and different ethnic population needs to be carried out to find out how this polymorphism imparts risk of obesity.

Our findings showed that obesity was more prevalent in females (57%) compared to males (43.4%). In addition, we found that factors such as physical exercise and a family history of obesity were important confounding factors. People with obesity had more comorbidities like hypertension and hypercholesterolemia. Women are more prone to be at a higher risk of being affected with obesity compared to men, which is supported to some extent by previous studies that reported women had a higher risk of being affected with obesity in the Iranian and Korean population but contradicts a study of the Lebanese population that showed men were more prone to become affected with obesity [23,24]. A study conducted on the Korean population revealed that the frequency of hypertension was higher in the group with obesity group [24] which is in alignment with our results. In addition, we observed that people with obesity had a family history of obesity but other studies did not confirm this association [16,23,24]. However, analysis of the SNPs with clinicopathological characteristics did not show any significant positive association.

Adequate vitamin D level is determined by measuring plasma levels of 25-hydroxyvitamin D (25[OH]-D) [14]. Our investigation demonstrates the notable difference in serum 25-hydroxyvitamin D levels between people with obesity and healthy controls. Serum 25-hydroxyvitamin D levels were significantly lower in participants with obesity (12.11 ± 1.64 ng/ml) than in healthy controls (33.71 ± 2.13 ng/ml) (P = 0.003). These results are in line with earlier studies that found obesity was linked to decreased vitamin D bioavailability and metabolic activity, possibly as a result of the vitamin’s sequestration in adipose tissue and changes in its metabolism [47]. Our finding of lower levels of serum vitamin D in people with obesity is in agreement with some previous studies. Studies in hospitalized individuals of Caucasian ancestry found that obesity was linked to decreased serum 25-hydroxyvitamin D (25D) levels [48–50]. Parikh et al. reported that adults with obesity exhibited lower levels of 25-hydroxyvitamin D (25-OH-D) and 1,25-dihydroxyvitamin D [17,51]. Similar outcomes were found in a study involving 2,126 patients conducted in Norway [51,52]. In our study, we found that, most of the people with obesity were deficient of vitamin D (65.3%) or insufficient (28.2%) which aligns with the findings of previous studies. However, a study suggested that individuals with obesity might spend less time exposing their skin to sunlight compared to individuals without obesity, which could reduce vitamin D synthesis [51,53]. A study by Wortsman et al. demonstrated that vitamin D, being fat-soluble, can become sequestered in adipose tissue, reducing its availability in the bloodstream. In their experiment, 19 lean (BMI ≤ 25 kg/m2) and 19 individuals with obesity (BMI > 30 kg/m2) were exposed to UVB irradiation for 24 hours. While baseline cholecalciferol levels were similar across groups, the participants with obesity showed a 57% lower serum cholecalciferol post-intervention compared to lean individuals. Both groups had comparable skin levels of 7-dehydrocholesterol, indicating that the lower bioavailability of synthesized cholecalciferol in the people with obesity group was due to sequestration in fat tissue [48,54]. This theory, supported by further evidence, explains why individuals with obesity often require 2–5 times more vitamin D to address or prevent deficiency [54,55]. It has been reported that serum 25D levels are inversely related to body fat content, with this relationship being stronger than those with BMI or body weight [51,56]. The first meta-analysis of the association between BMI and vitamin D deficiency emphasizes the significant incidence of vitamin D insufficiency in persons with overweight issue and obesity. Regardless of age, vitamin D deficiency was 35% more common in people with obesity than in eutrophic people, and 24% higher than in people with overweight issue. 37% of children and adolescents with obesity lacked vitamin D, compared to 33% of adults and the elderly with obesity [14,57]. Moreover, vitamin D insufficiency was linked to obesity in both Asians and European Americans [57,58].

Furthermore, we found that serum VDR levels were considerably lower in the people with obesity group compared to the healthy control group (P < 0.001). Decreased VDR levels in people with obesity might be the result of compromised receptor-mediated signaling, which could worsen the negative effects of vitamin D insufficiency on metabolic health [59]. VDR mRNA expression was higher in visceral and subcutaneous adipose tissues in those with morbidity obesity compared to non-obese individuals [60,61]. It is unclear if upregulation of gene expression in adipose tissue results in upregulation of VDR protein expression. Due to the lack of gene expression data of VDR in our study, we cannot conclude whether VDR mRNA expression is upregulated or downregulated in serum in those with obesity and how it relates to the downregulation of serum VDR protein level in obesity.

In addition, we discovered no cis-regulatory influence of rs7975232 and rs731236 on vitamin D protein level. Genotyping more SNPs is necessary to uncover possible variants that may alter vitamin D level and serve as eQTLs for obesity-related and circulating biomarkers.

There were some limitations in our study. Although BMI is widely used as a standard parameter to classify obesity, it has several limitations. For instance, it fails to distinguish between lean mass and fat mass, nor does it include data on the distribution of fat (central versus peripheral), which is essential for determining health risks. Additionally, its application may differ among various populations and ethnic groupings. We were unable to analyze other anthropometric parameters, such as waist circumference (WC), waist-to-hip ratio (WHR), and waist-to-height ratio (WHtR). These inherent limitations of BMI may lead to differences in obesity prevalence observed when comparing our findings to those from other populations or studies that used different or more extensive assessment methodologies. Our sample size was relatively small, which would only be able to detect medium to large effects. Our findings indicated a higher prevalence of obesity in females (57%) compared to males (43.4%), consistent with national data reporting higher obesity rates in Bangladeshi females [5,6]. Moreover, the study may have limited power to detect a significant genotype-disease association. Due to the absence of gene expression data, we could not ascertain whether the SNP polymorphism has any cis-regulatory influence on gene expression. Genotyping more SNPs is warranted to find the putative polymorphisms of the VDR gene that increase the risk of obesity.

### Conclusions

Despite the limitations, ours is the first case-control study that we are aware of that has identified the association between VDR gene polymorphisms, ApaI (rs7975232), with obesity in the Bangladeshi population, along with lower serum 25-hydroxy vitamin D level and lower serum VDR levels. Therefore, future studies should incorporate larger sample sizes and a more diverse range of ethnicities to validate our findings.

## Supporting information

S1 File Raw images of gel electrophoresis. (PDF)

S2 File Minimal data set. (XLSX)
