# Supplementary figures and images for: Downregulation of serum vitamin D receptor level, serum 25-hydroxyvitamin D, and association of vitamin D receptor gene polymorphisms ApaI and TaqI with obesity in the Bangladeshi population
Source: PLoS One. 2025 Nov 20;20(11):e0337523. doi: 10.1371/journal.pone.0337523 (PMC12633872; doi:10.1371/journal.pone.0337523)

CA CC CA AA CA CA AA AA

800 bp →  
700 bp →  
600 bp →  
500 bp →  
400 bp →  
300 bp →  
200 bp →  
100 bp →

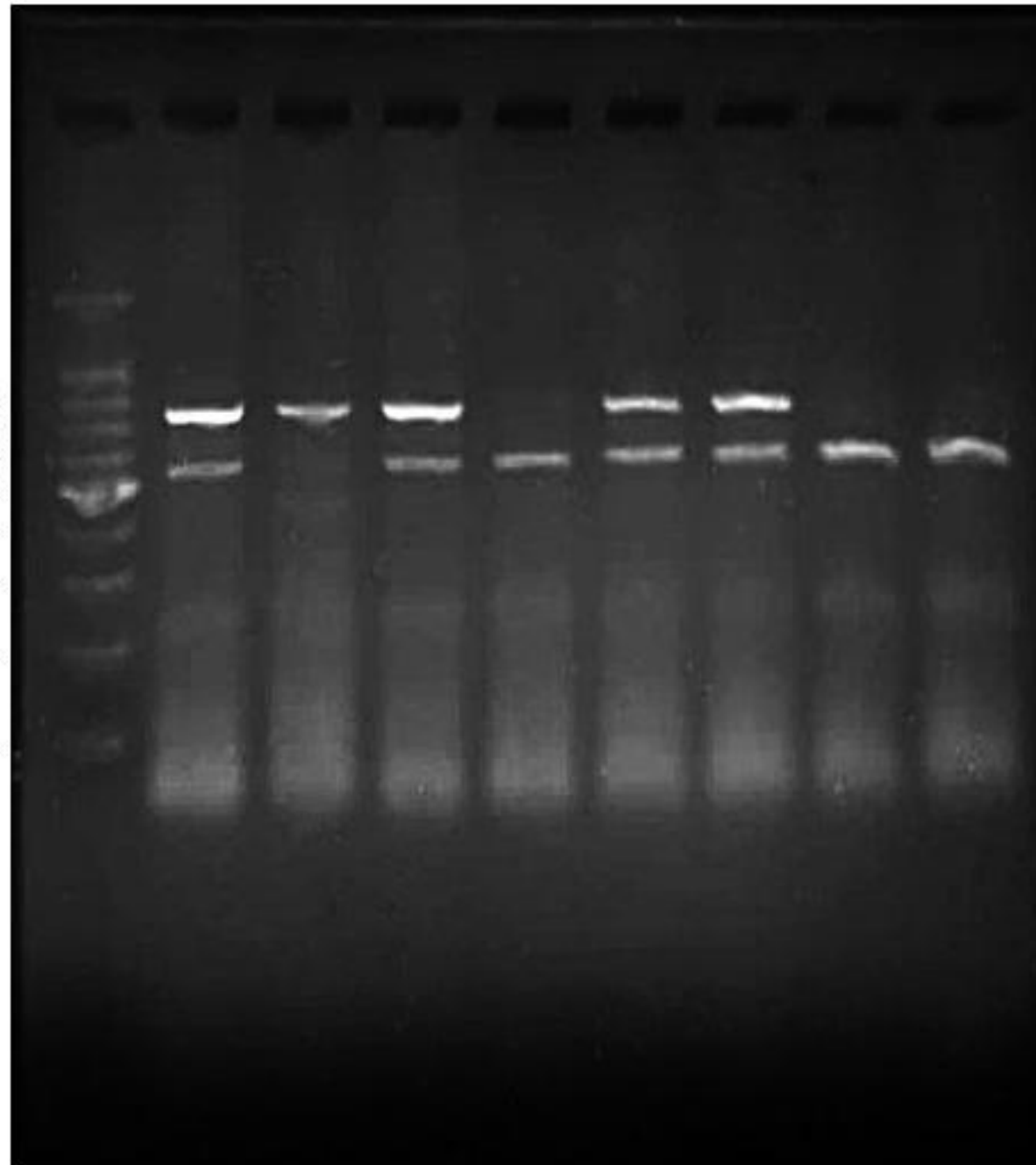

← 745 bp  
← 528 bp

TT TT TC TC CC TT

600 bp →  
500 bp →  
400 bp →  
300 bp →  
200 bp →  
100 bp →

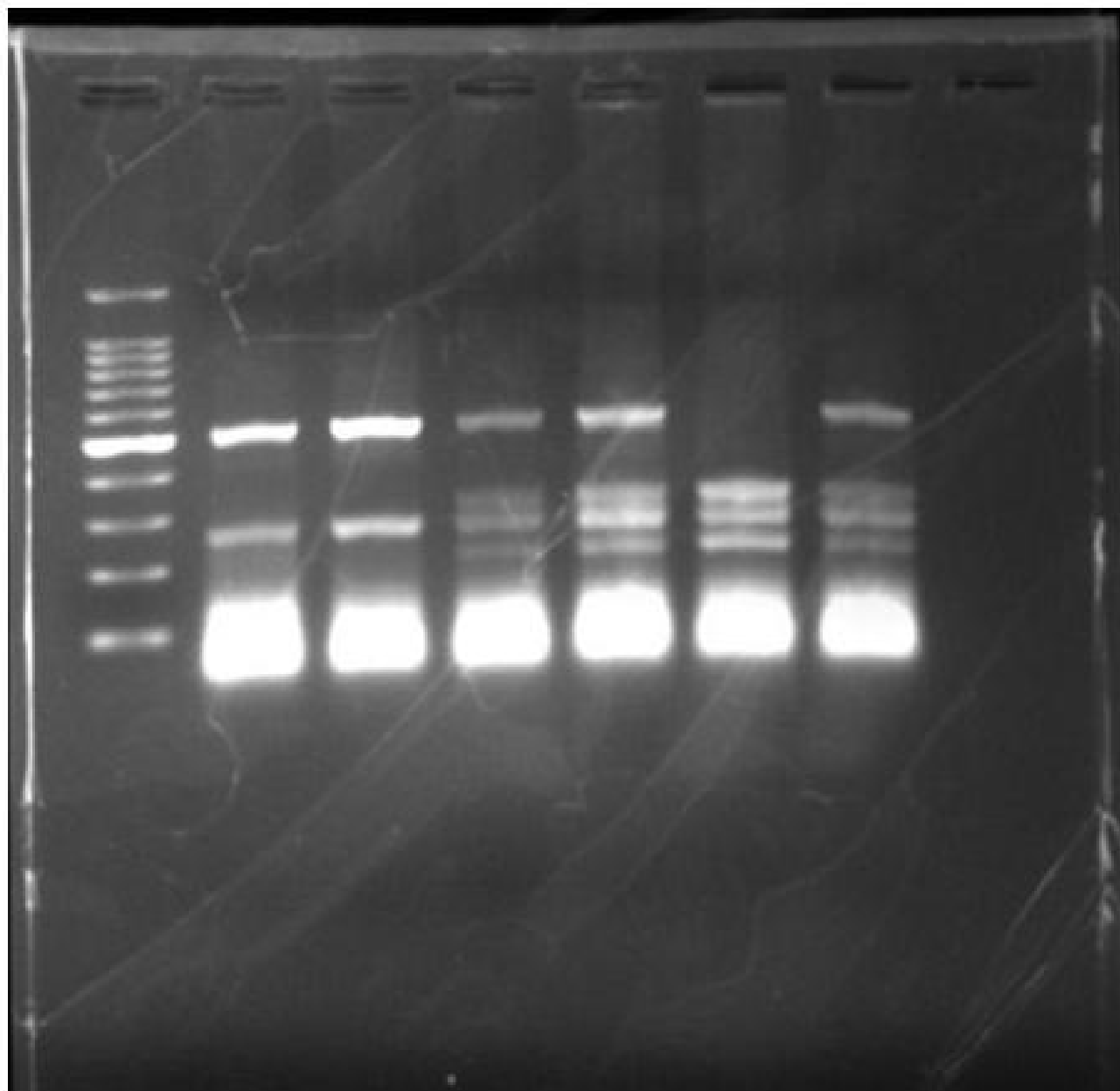

← 495 bp  
← 290 bp  
← 245 bp  
← 205 bp

Supplement: S1 File — (PDF) [file pone.0337523.s001.pdf]
